# Associations of childhood and adult socioeconomic circumstances with recommended food habits among young and midlife Finnish employees

**DOI:** 10.1186/s40795-022-00557-0

**Published:** 2022-07-14

**Authors:** Jatta Salmela, Anne Kouvonen, Elina Mauramo, Ossi Rahkonen, Eva Roos, Tea Lallukka

**Affiliations:** 1 Department of Public Health, University of Helsinki, PO Box 20 (Tukholmankatu 8 B), 00014 Helsinki, Finland; 2 Faculty of Social Sciences, University of Helsinki, Helsinki, Finland; 3 Centre for Public Health, Queen’s University Belfast, Belfast, UK; 4 Department of Food Studies, Nutrition and Dietetics, Uppsala University, Uppsala, Sweden; 5 Folkhälsan Research Center, Helsinki, Finland

**Keywords:** Employees, Food habits, Nutrition recommendations, Public sector, Socioeconomic circumstances

## Abstract

**Background:**

Disadvantaged socioeconomic circumstances are associated with certain unhealthy food habits such as inadequate consumption of fruit and vegetables. This study examined whether multiple socioeconomic measures are consistently associated with a variety of food habits.

**Methods:**

We examined associations of 2 childhood and 6 adult socioeconomic measures with 8 recommended food habits among 19–39-year-old employees of the City of Helsinki, Finland. The data were collected in 2017 via online and mailed surveys. Our sample consisted of 4621 employees (80% women). The analyses included adjusted binary logistic regression models.

**Results:**

More advantaged socioeconomic circumstances were positively associated with the recommended consumption of vegetables, fruit or berries, dark bread, skimmed milk products, fish, and cooking oil, but not consistently with red or processed meat and fat spread. All socioeconomic measures were positively associated with having several (6–8) recommended food habits after gender and age adjustments. The strongest associations were found for participant’s education, occupational class, and current financial difficulties. These associations remained after adjustments of childhood and adult socioeconomic measures, although especially participant’s education attenuated the associations for occupational class.

**Conclusions:**

The consistent associations between multiple childhood and adult socioeconomic measures and food habits found among employees highlight the need for improving food habits among people with disadvantaged socioeconomic circumstances in particular. Financial barriers together with social aspects of adhering to healthy diets should be considered in future dietary interventions and policy actions.

**Supplementary Information:**

The online version contains supplementary material available at 10.1186/s40795-022-00557-0.

## Introduction

Socioeconomic differences in adults’ nutrition and diets have been broadly observed in Europe over several decades [1–5]. Disadvantaged socioeconomic circumstances (SEC) have been associated with unhealthier overall diet quality, measured by dietary scores, indices, and patterns [4, 6–8]. Among single food habits, disadvantaged SEC have been associated with increased consumption of butter, added fats, refined grains, meat and meat products, as well as decreased consumption of fish, low-fat dairy products, and, in particular, fruit and vegetables [3, 7, 9–11]. Furthermore, reduced fibre and micronutrient intake, such as iron, vitamin C, and vitamin D, have been observed among individuals with disadvantaged SEC [5, 7]. However, there are some unhealthy foods, such as cheese, candies, and pastries, that are consumed more commonly among individuals with advantaged SEC [2, 7, 11, 12]. Although women’s food habits are known to be healthier than men’s in general, no clear gender differences in the direction and magnitude of socioeconomic differences in food habits have been found [4, 10].

Given that socioeconomic differences in diet-related diseases are well established—more disadvantaged SEC being associated with an increased risk of obesity, type 2 diabetes, and cardiovascular diseases, for example [10, 13, 14]—disparities in food habits should be intervened on to tackle larger health disparities. In Finland, whilst population health in general has improved, health disparities between socioeconomic groups have persisted and stayed relatively wide, even though reducing health disparities has been a key national health policy goal during past decades [15, 16]. Similarly, socioeconomic differences in food habits persist in Finland. The National FinDiet 2017 Survey showed that adults with higher educational level consumed more fruit, vegetables, and vegetable oils, and less processed meat products than those with the lowest educational level [17]. Additionally, the adults with higher educational level received more several beneficial nutrients from their diets, such as polyunsaturated fatty acids, fibre, folate, and vitamin C and D, and less, for instance, sodium and saturated fatty acids, than those with lower educational levels [17].

A common explanation for socioeconomic differences in food habits relates to the cost of foods: unhealthier, energy-dense, and nutritionally poor foods tend to cost less than the healthier ones [18, 19]. Individuals with disadvantaged SEC are more likely to suffer from financial difficulties, which make them more vulnerable to unhealthier food consumption [4, 20]. Low-cost and energy-dense foods are also potentially more easily available in socioeconomically deprived areas and neighbourhoods [7]. The contribution of higher education to healthier food habits, in turn, is largely explained by nutrition knowledge and beliefs, and the ability to adopt health information [6, 7]. The motives for food selection also differ between socioeconomic groups: individuals in disadvantaged SEC are more likely to prefer foods that are familiar and cheap than those in advantaged SEC [21]. The few opposite findings where advantaged SEC have been associated with unhealthier dietary patterns, such as increased consumption of cheeses, have been explained by the ‘modernity hypothesis’, wherein individuals with advantaged SEC consume more foods that are culturally and socially eminent or trendy [11]. Overall, the associations between SEC and diet partially reflect more general socioeconomic differences in society, including socioeconomic differences in other health behaviours [8].

Most existing studies have used either education, occupational class, or income as a SEC measure [5]. Mostly, these measures have yielded parallel associations with diet, although they also reflect different underlying social processes and are not fully interchangeable [22, 23]. The impacts of different SEC measures on health are partially explained or mediated by other SEC measures [24]. For instance, a US study observed that the pathway from income to diet quality via diet cost was moderated by education [18]. Furthermore, considering a wider scope of SEC measures can reveal some unique aspects of SEC. For instance, experienced financial difficulties can more closely capture everyday challenges in buying foods and managing dietary behaviours than income [4]. Additionally, the majority of existing studies have focused on adult SEC (i.e., present SEC), neglecting the potential contribution of childhood SEC (i.e., past SEC, such as parental education) to dietary differences in adults [9, 10]. Thus, simultaneous use of multiple past and present SEC measures enables us to observe their interrelations, and consequently, to broaden our understanding on how SEC are associated with adults’ food habits [22].

A previous study by our research group showed that more advantaged adult SEC, in terms of education, occupational class, household income, home ownership, and financial difficulties, were associated with having recommended food habits among 40–60-year-old Finnish municipal employees [25]. Childhood SEC were not associated with food habits [25]. As observed in other studies [4, 7], the associations between SEC and food habits were gradual rather than threshold-dependent. Since our previous study was based on data on midlife and older employees in 2000–2002, updated knowledge is needed concerning younger and current employees due to potential age and cohort effects. For example, socioeconomic living conditions may be different in younger than in older employees. Additionally, our previous study showed that employees from a younger birth cohort were more likely to gain weight than employees from an older birth cohort [26]. Updated knowledge about socioeconomic differences in food habits can help to intervene on these unfavourable trends in diet-related disparities [8]. Thus, this study aimed to examine associations between multiple childhood and adult SEC and recommended food habits among 19–39-year-old Finnish municipal employees. The key sociodemographic were considered in the analyses.

## Methods

### Data and participants

The data were from the Helsinki Health Study, consisting of 19–39-year-old employees of the City of Helsinki, Finland [27]. The study gathers a variety of questions on employees’ health and wellbeing and their sociodemographic and work-related determinants. Online and mailed surveys were sent in autumn 2017 to all those who had been employed for at least 4 months with a contract of 50% or more (*N* = 11,459, i.e., the target population) [27]. Telephone interviews with a shortened survey version were conducted among the non-respondents. The final response rate was 51.5% (*n* = 5898); 78.5% were women. This study excluded telephone interviewees (*n* = 787) since limited information on socioeconomic characteristics and food habits were available from these interviews. Additionally, respondents with missing data on gender (*n* = 1), age (*n* = 11), and any food habits (*n* = 923) and SEC measures (*n* = 1161) of interest were excluded. Consequently, the final analytical sample consisted of 4621 participants, of which 79.5% were women. More detailed inclusion criteria are shown in Fig. S1 (Additional file 1).

The Helsinki Health Study protocol has been approved by the ethics committees of the Department of Public Health, the University of Helsinki, and the health authorities of the City of Helsinki. All study participants gave informed consent prior to their inclusion in the study.

### Outcome measures: food habits

The Helsinki Health Study survey included a 14-item food frequency questionnaire (FFQ) where participants were asked how often they had consumed each food item during the past 4 weeks. The response alternatives were: not at all, 1–3 times a month, once a week, 2–4 times a week, 5–6 times a week, once a day, or twice a day or more. Six food items were used from the FFQ, and were dichotomised into ‘recommended’ and ‘other’ food habits based on the Finnish nutrition and food recommendations [28]. The recommended food habits were: 1) fresh or cooked vegetables at least twice a day, 2) fruit or berries at least twice a day, 3) dark (wholegrain) bread at least once a day, 4) skimmed milk products at least once a day, 5) fish at least 2–4 times a week, and 6) red or processed meat products 2–4 times a week at most. Additionally, participants were inquired about the type of fat they mostly use 7) on bread (3 response alternatives) and 8) in cooking (6 response alternatives). Vegetable-based margarine on bread and vegetable-based margarine or oil in cooking were categorised as ‘recommended’ and other alternatives as ‘other’.

The Finnish nutrition and food recommendations in terms of the used food habit measures are as follows: 1–2) at least 500 g a day of vegetables, fruit, berries, and mushrooms, of which about one half should consist of fruit and berries and the rest of vegetables; 3) cereal products of 6 and 9 portions for women and men, respectively, of which at least one half should be whole grain cereals (one portion equals one slice of bread); 4) 5–6 dl of liquid fat-free or low-fat milk products; 5) fish 2–3 times a week; 6) 500 g red or processed meat (as cooked) a week at most; and 7–8) the use of vegetable-based oils and spreads (excluding coconut oil and palm oils) on bread and in cooking [28]. Since the FFQ used in this study could not capture the amounts of consumed foods in detail, the classifications were made so that they would as closely as possible correspond to the current nutrition and food recommendations.

We summed the recommended food habits (1 point for recommended and 0 points for other), which yielded a sum score ranging 0–8 points. Adapting the procedure used in previous studies [25, 29] and taking into consideration the feasibility of group sizes, receiving 6–8 points was categorised as ‘having several recommended food habits’ and 0–5 points as ‘other’. Additionally, consuming fresh or cooked vegetables, or fruit or berries at least twice a day was required to belong to the group of having several recommended food habits. This was done to highlight the importance of adequate consumption of fruit and vegetables as healthy dietary habits [28, 30, 31]. The correlations between single food items varied between − 0.08 and 0.40. The highest positive correlation was found between fresh or cooked vegetables, and fruit or berries. Fat spread, dark bread, and skimmed milk products showed negative correlations (from − 0.02 to − 0.08) with red or processed meat.

### Exposure measures: childhood and adult socioeconomic circumstances

We used eight measures of childhood and adult SEC. Parental education was based on the highest educational attainment of participants’ mother or father, including four response alternatives. We categorised the variable into three groups: higher education, upper secondary school, and vocational school or less. Childhood financial difficulties were inquired retrospectively by asking whether the respondent’s family experienced substantial financial difficulties during the respondent’s childhood or adolescence, before the age of 16 (‘yes’/‘no’).

The participant’s own education included six response alternatives, which we categorised into three groups: master’s degree or higher, bachelor’s degree, and upper secondary school. We categorised occupational class into four groups: managers and professionals, semi-professionals, routine non-manuals, and manual workers [32]. Household income was inquired by a question of typical monthly net income with 10 income-level alternatives. Using the OECD (Organisation for Economic Co-operation and Development) equivalence scale for weighting, the respondent received the value of 1.0, other adults 0.5, and children 0.3 [33], and the weighted household income was divided into quartiles: 2809–7040, 2333–2800, 1768–2300, and 360–1750 (€/month). We dichotomised housing tenure into owner-occupiers and renters/others. Current financial difficulties were inquired by two questions [34]: ‘How often do you not have enough money to buy the kind of food or clothing you or your family need?’ and ‘How much difficulty do you have in meeting the payment of bills?’. Five response alternatives were given for both questions, ranging from ‘always’ to ‘never’ and from ‘very little or not at all’ to ‘very much’, respectively. We categorised ‘always’ or ‘often’ having money to buy food and clothing and ‘very little or not at all’ or ‘little’ difficulties in paying bills into having no or few financial difficulties, while the rest were categorised into having financial difficulties [35]. Household wealth was measured with a question inquiring about the amount of money the respondent would have if all household assets were cashed and all debts paid off. We categorised wealth into three groups: ≥ 100,000€; 10,000–99,999€; and < 10,000€.

### Covariates

Gender included alternatives woman/man. Age was dichotomised into 30–39-year-olds and 19–29-year-olds, given that dietary habits among adult Finns vary between age groups [17, 36]. Immigrant background has been shown to influence dietary habits in Finland, and the associations are socioeconomically patterned [37]. Thus, we dichotomised country of birth into born in Finland or elsewhere, and participants with missing data (*n* = 31) were merged with the participants born in Finland to avoid exaggerated differences between groups (conservative way of handling missing data). Marital status is known to be associated with dietary habits as well [38], thus we dichotomised it into married/cohabiting and others. Participants with missing data on the variable (*n* = 10) were merged with married/cohabiting participants. Finally, participants were dichotomised into those who had 0–18-year-old children in their household and those who did not have, given that parenthood potentially affects SEC–diet associations [39].

We considered only key sociodemographic factors in the analyses and left out potential explanatory factors, such as lifestyle and health variables, because the primary aim of this study was not to examine which variables explain socioeconomic differences in food habits. However, we performed a supplementary analysis where we examined the contribution of BMI and long-term illness to the main findings (Table S1). These factors did not contribute to the findings.

### Statistical methods

All analyses were performed using Stata statistical software package version 16 (StataCorp LLC, College Station, TX, USA). We examined the associations between SEC and food habits using binary logistic regression analyses. In Model 1, we adjusted the analyses for gender and age, and in Model 2, further for country of birth, marital status, and having children in the household. In Model 3, we adjusted the analyses further for childhood SEC (parental education and childhood financial difficulties). Lastly, in Model 4, we added conventional adult SEC measures (participant’s own education, occupational class, and household income) to Model 2. We did not to include all adult SEC measures in the models since high correlations were found between some measures (Table S2, Additional file 1). The highest correlations were found between participant’s own education and occupational class (> 0.7), and between housing tenure and household wealth (> 0.5). However, we found no indication for multi-collinearity (variance inflation factor [VIF] ranged 1.04–2.76) (Table S2, Additional file 1).

Since the proportion of men was small in our study, we analysed women and men together and ran corresponding gender-specific analyses as supplementary analyses (Tables S3–S6, Additional file 1). We tested gender interactions with all SEC measures using the dichotomous ‘several recommended food habits’ variable as an outcome. We did not find any statistically significant (*p* < 0.05) associations for interaction variables, which supported analysing women and men together. Additionally, we performed gender-adjusted supplementary analyses by age groups (19–29 years vs. 30–39 years) (Table S7), given that our SEC measures may reflect different aspects of SEC for those close to 20 years old than those close to 40 years old. Because the small group sizes in some SEC measures limited the interpretation of these findings and because the associations were parallel between the age groups, we decided to use age only as a covariate in our main analyses.

## Results

The prevalence of having single recommended food habits varied remarkably between the food items (Table 1). While the majority of participants met the recommended food habit criteria for red or processed meat (81%) and cooking fat (70%), other foods were consumed less often than recommended. In particular, consuming fish at least 2–4 times a week was scarce among participants (27%). Additionally, only 35% of women and 18% of men consumed fruit or berries as recommended. Overall, 16% of women and 8% of men had several [6–8] recommended food habits. More details about the distribution of having recommended food habits among the participants are shown in Table S8 (Additional file 1).

**Table 1:** Recommended food habits ^a^ among the study participants

	All, n (%)	Women, n (%)	Men, n (%)
**Single food habits**			
Fresh or cooked vegetables at least twice a day	2205 (47.7)	1926 (52.4)	279 (29.6)
Fruit or berries at least twice a day	1470 (31.8)	1303 (35.4)	167 (17.7)
Dark bread daily	1863 (40.3)	1539 (41.9)	324 (34.3)
Skimmed milk products daily	1638 (35.5)	1386 (37.7)	252 (26.7)
Fish at least 2–4 times/week	1232 (26.7)	967 (26.3)	265 (28.1)
Red or processed meat 2–4 times/week at most	3763 (81.4)	3070 (83.5)	693 (73.4)
Vegetable-based margarine on bread	1642 (35.5)	1296 (35.3)	346 (36.7)
Vegetable-based margarine or oil in cooking	3236 (70.0)	2597 (70.6)	639 (67.7)
**Number of recommended food habits**			
0–5 (‘other’)	3945 (85.4)	3073 (83.6)	872 (92.4)
6–8 (‘having several recommended food habits’) ^b^	676 (14.6)	604 (16.4)	72 (7.6)

^a^ Food habits were dichotomised into ‘recommended’ and ‘other’ food habits based on the Finnish nutrition and food recommendations [24]

^b^ Consuming fresh or cooked vegetables, or fruit or berries at least twice a day was required for belonging to this group

Having several recommended food habits was more common among individuals with more advantaged SEC (Table 2), regardless of the SEC measure. A consistent socioeconomic gradient could be seen in all SEC measures that included more than two hierarchical classes. However, in even the most advantaged socioeconomic groups at most only one-fifth of the participants had several recommended food habits. Older participants, those born in Finland, those married or cohabiting, and those with children in the household were more likely to have several recommended food habits.

**Table 2:** Characteristics of the study population by socioeconomic and dietary factors

	All, n (%)	Having several recommended food habits ^a^, n (%)
**Total, n (%)**	**4621 (100)**	**676 (14.6)**
***Childhood socioeconomic measures***		
**Parental educational level**		
Vocational school or lower	2040 (44.2)	258 (12.6)
Upper secondary school	580 (12.6)	81 (14.0)
Higher education	2001 (43.3)	337 (16.8)
**Childhood financial difficulties**		
Yes	997 (21.6)	116 (11.6)
No	3624 (78.4)	560 (15.5)
***Adult socioeconomic measures***		
**Educational level**		
Upper secondary school or lower	1542 (33.4)	141 (9.1)
Bachelor’s degree	1709 (37.0)	258 (15.1)
Master’s degree or higher	1370 (29.7)	277 (20.2)
**Occupational class**		
Manual worker	237 (5.1)	14 (5.9)
Routine non-manual worker	1225 (26.5)	130 (10.6)
Semi-professional	1872 (40.5)	280 (15.0)
Manager or professional	1287 (27.9)	252 (19.6)
**Household income**		
Lowest quartile	1164 (25.2)	144 (12.4)
2nd lowest quartile	1421 (30.8)	185 (13.0)
2nd highest quartile	1147 (24.8)	177 (15.4)
Highest quartile	889 (19.2)	170 (19.1)
**Housing tenure**		
Renter or other	2465 (53.3)	294 (11.9)
Owner-occupier	2156 (46.7)	382 (17.7)
**Financial difficulties**		
Yes	1014 (21.9)	87 (8.6)
No or few	3607 (78.1)	589 (16.3)
**Household wealth**		
< 10,000€	1611 (34.9)	171 (10.6)
10,000–99,999€	1875 (40.6)	285 (15.2)
≥ 100,000€	1135 (24.6)	220 (19.4)
***Sociodemographic factors***		
**Age**		
19–29 years	1479 (32.0)	155 (10.5)
30–39 years	3142 (68.0)	521 (16.6)
**Country of birth**		
Other	284 (6.2)	31 (10.9)
Finland	4337 (93.9)	645 (14.9)
**Marital status**		
Other	1537 (33.3)	186 (12.1)
Married or co-habiting	3084 (66.7)	490 (15.9)
**Having children in the household**		
No	2752 (59.6)	326 (11.8)
Yes	1869 (40.5)	350 (18.7)

^a^ The share of participants within each socioeconomic group who had several recommended food habits. Having 6–8 recommended food habits and consuming fresh or cooked vegetables, or fruit or berries at least twice a day was required for belonging to this group

Having a single recommended food habit was more common in socioeconomically more advantaged groups, regardless of the SEC measure (Table 3). When adjusting for gender and age, the highest odds were found for participant’s own occupational class with vegetable consumption (e.g., for managers and professionals, odds ratio [OR] 2.91, 95% confidence interval [CI] 2.09–4.03), participant’s own educational level with cooking fat (e.g., for participants with master’s degree or higher, OR 2.16, 95% CI 1.83–2.55), and participant’s own occupational class with meat consumption (e.g., for managers and professionals, OR 2.03, 95% CI 1.46–2.82). The direction of the associations between SEC measures and fat spread varied depending on the SEC measure used, and the associations were mostly statistically non-significant. Additionally, being an owner-occupier was negatively associated with the recommended consumption of red or processed meat. Participant’s own educational level showed stronger associations with single food habits than parental educational level, and these patterns could mostly be seen for current financial difficulties versus childhood financial difficulties as well.

**Table 3:** Gender- and age-adjusted associations between socioeconomic circumstances and single recommended food habits

	Odds ratios (95% confidence intervals)							
	Fresh or cooked vegetables at least twice a day	Fruit or berries at least twice a day	Dark bread daily	Skimmed milk products daily	Fish at least 2–4 times/week	Red or processed meat 2–4 times/week at most	Vegetable-based margarine on bread	Vegetable-based margarine or oil in cooking
***Childhood socioeconomic measures***								
**Parental educational level** ^a^								
Upper secondary school	1.22 (1.01–1.47)	1.12 (0.91–1.37)	1.02 (0.84–1.24)	1.02 (0.84–1.24)	1.00 (0.81–1.25)	1.39 (1.08–1.79)	0.87 (0.71–1.06)	1.25 (1.02–1.53)
Higher education	1.43 (1.26–1.63)	1.28 (1.12–1.46)	1.02 (0.90–1.16)	1.14 (1.00–1.29)	1.43 (1.25–1.65)	1.25 (1.07–1.47)	0.90 (0.79–1.03)	1.41 (1.23–1.61)
**Childhood financial difficulties** ^b^								
No	1.00 (0.95–1.27)	1.19 (1.02–1.40)	1.12 (0.97–1.29)	1.20 (1.04–1.40)	1.17 (0.99–1.38)	1.12 (0.94–1.34)	1.12 (0.94–1.34)	1.01 (0.87–1.18)
***Adult socioeconomic measures***								
**Educational level** ^c^								
Bachelor’s degree	1.52 (1.32–1.75)	1.54 (1.32–1.80)	1.24 (1.08–1.43)	1.23 (1.06–1.42)	1.26 (1.07–1.49)	1.15 (0.97–1.37)	0.86 (0.74–0.99)	1.68 (1.45–1.95)
Master’s degree or higher	1.90 (1.63–2.21)	1.78 (1.50–2.10)	1.06 (0.91–1.23)	1.42 (1.22–1.67)	1.78 (1.50–2.11)	1.73 (1.41–2.11)	0.77 (0.66–0.90)	2.16 (1.83–2.55)
**Occupational class** ^d^								
Routine non-manual worker	1.63 (1.17–2.27)	1.06 (0.75–1.50)	1.33 (0.98–1.81)	1.22 (0.88–1.69)	0.98 (0.70–1.38)	1.25 (0.90–1.72)	0.86 (0.64–1.14)	0.80 (0.60–1.08)
Semi-professional	2.19 (1.59–3.03)	1.28 (0.92–1.80)	1.48 (1.10–2.01)	1.51 (1.10–2.08)	1.18 (0.85–1.64)	1.42 (1.04–1.95)	0.77 (0.58–1.03)	1.32 (0.99–1.77)
Manager or professional	2.91 (2.09–4.03)	1.53 (1.08–2.15)	1.31 (0.97–1.78)	1.60 (1.16–2.21)	1.57 (1.13–2.19)	2.03 (1.46–2.82)	0.71 (0.53–0.94)	1.65 (1.22–2.24)
**Household income** ^e^								
2nd lowest quartile	1.09 (0.93–1.28)	1.15 (0.97–1.37)	1.01 (0.86–1.18)	1.06 (0.90–1.25)	0.98 (0.81–1.17)	1.15 (0.94–1.40)	1.00 (0.85–1.18)	1.01 (0.86–1.20)
2nd highest quartile	1.41 (1.19–1.66)	1.37 (1.15–1.64)	1.06 (0.90–1.25)	1.08 (0.90–1.28)	1.08 (0.90–1.31)	1.20 (0.97–1.48)	0.89 (0.75–1.06)	1.46 (1.22–1.75)
Highest quartile	1.66 (1.38–1.98)	1.48 (1.22–1.79)	0.97 (0.81–1.16)	1.33 (1.11–1.60)	1.42 (1.17–1.73)	1.23 (0.98–1.54)	0.92 (0.77–1.11)	1.41 (1.16–1.71)
**Housing tenure** ^f^								
Owner-occupier	1.33 (1.18–1.50)	1.10 (0.97–1.26)	1.20 (1.06–1.36)	1.26 (1.11–1.43)	1.20 (1.04–1.37)	0.77 (0.66–0.91)	1.05 (0.92–1.19)	1.20 (1.05–1.37)
**Financial difficulties** ^g^								
No or few	1.47 (1.27–1.70)	1.42 (1.21–1.66)	1.25 (1.08–1.44)	1.17 (1.00–1.35)	1.34 (1.13–1.58)	1.17 (0.98–1.40)	1.02 (0.88–1.18)	1.75 (1.51–2.02)
**Household wealth** ^h^								
10,000–99,999€	1.23 (1.07–1.41)	1.23 (1.06–1.42)	1.16 (1.01–1.33)	1.17 (1.02–1.35)	1.19 (1.01–1.39)	0.95 (0.79–1.13)	0.99 (0.86–1.13)	1.31 (1.14–1.52)
≥ 100,000€	1.52 (1.30–1.79)	1.29 (1.09–1.53)	1.22 (1.04–1.43)	1.54 (1.31–1.81)	1.62 (1.36–1.93)	0.84 (0.69–1.02)	1.18 (1.00–1.38)	1.50 (1.26–1.78)

Reference groups: ^a^ vocational school or lower, ^b^ yes, ^c^ upper secondary school or lower, ^d^ manual worker, ^e^ lowest quartile, ^f^ renter or other, ^g^ yes, ^h^ < 10,000€

When examining food habits together, each SEC measure was positively associated with having several [6–8] recommended food habits (Table 4). Participant’s own educational level, occupational class, and current financial difficulties showed the strongest associations after adjusting for gender and age (Model 1). Further adjustment for country of birth, marital status, and having children in the household did not affect the associations (Model 2). Additionally, childhood SEC (i.e., parental education and childhood financial difficulties) only slightly attenuated the associations (Model 3). The associations between occupational class and having several recommended food habits, however, were more broadly explained by other conventional adult SEC measures: that is, educational level and—to a lesser extent upon further examination—household income (Model 4). Although adult SEC showed stronger positive associations with having several recommended food habits than childhood SEC, higher parental education and not having financial difficulties in childhood remained statistically significantly associated with having several recommended food habits even after adjustment of conventional adult SEC measures (Model 4).

**Table 4:** Associations between socioeconomic circumstances and having several recommended food habits ^a^

	Odds ratios (95% confidence intervals)			
	M1: Gender and age adjustments	M2: M1 + country of birth, marital status, and having children in household	M3 ^b^: M2 + parental educational level and childhood financial difficulties	M4 ^b^: M2 + own education, occupational class, and household income
***Childhood socioeconomic measures***				
**Parental educational level** ^c^				
Upper secondary school	1.21 (0.92–1.58)	1.19 (0.91–1.57)	1.18 (0.90–1.55)	1.12 (0.85–1.47)
Higher education	1.49 (1.25–1.78)	1.51 (1.26–1.80)	1.46 (1.22–1.75)	1.27 (1.05–1.53)
**Childhood financial difficulties** ^d^				
No	1.42 (1.15–1.76)	1.41 (1.14–1.75)	1.33 (1.07–1.66)	1.30 (1.05–1.62)
***Adult socioeconomic measures***				
**Educational level** ^e^				
Bachelor’s degree	1.60 (1.28–1.99)	1.60 (1.28–2.00)	1.53 (1.22–1.91)	1.48 (1.10–2.00)
Master’s degree or higher	2.19 (1.75–2.74)	2.22 (1.77–2.78)	1.99 (1.58–2.53)	1.85 (1.29–2.67)
**Occupational class** ^f^				
Routine non-manual worker	1.45 (0.82–2.59)	1.46 (0.82–2.60)	1.45 (0.81–2.59)	1.50 (0.84–2.67)
Semi-professional	2.06 (1.17–3.62)	2.03 (1.16–3.58)	1.95 (1.11–3.43)	1.52 (0.84–2.76)
Manager or professional	2.80 (1.59–4.93)	2.80 (1.59–4.93)	2.51 (1.42–4.43)	1.61 (0.86–3.00)
**Household income** ^g^				
2nd lowest quartile	1.07 (0.84–1.35)	1.21 (0.95–1.55)	1.17 (0.92–1.49)	1.06 (0.83–1.36)
2nd highest quartile	1.31 (1.03–1.67)	1.42 (1.10–1.84)	1.35 (1.04–1.74)	1.18 (0.91–1.54)
Highest quartile	1.61 (1.26–2.06)	1.85 (1.43–2.40)	1.71 (1.31–2.22)	1.41 (1.07–1.86)
**Housing tenure** ^h^				
Owner-occupier	1.44 (1.21–1.71)	1.30 (1.09–1.56)	1.25 (1.04–1.50)	1.14 (0.95–1.38)
**Financial difficulties** ^i^				
No or few	2.12 (1.67–2.69)	2.14 (1.68–2.73)	2.03 (1.59–2.59)	1.81 (1.41–2.33)
**Household wealth** ^j^				
10,000–99,999€	1.53 (1.23–1.86)	1.47 (1.19–1.81)	1.40 (1.13–1.73)	1.30 (1.05–1.62)
≥ 100,000€	1.88 (1.51–2.35)	1.72 (1.36–2.17)	1.56 (1.22–1.98)	1.37 (1.07–1.76)

^a^ Having 6–8 recommended food habits and consuming fresh or cooked vegetables, or fruit or berries at least twice a day was required for belonging to this group. Comparison group: having 0–5 recommended food habits

^b^ Mutual adjustment has been performed in Models 3 and 4 in the cases where there are same variables both as an exposure and as a covariate. For example, concerning parental educational level as an exposure measure in Model 3, the analysis is adjusted for Model 2 covariates together with childhood financial difficulties

Reference groups: ^c^ vocational school or lower, ^d^ yes, ^e^ upper secondary school or lower, ^f^ manual worker, ^g^ lowest quartile, ^h^ renter or other, ^i^ yes, ^j^ < 10,000€

## Discussion

### Main findings of the study

This study examined the associations of childhood and adult SEC with recommended food habits among 19–39-year-old Finnish municipal employees. Both childhood and adult SEC were positively associated with the recommended consumption of fresh or cooked vegetables, fruit or berries, dark bread, skimmed milk products, fish, and cooking oil, but inconsistently associated with red or processed meat and fat spread. When investigating all eight food habits together, we observed clear socioeconomic gradients in having several [6–8] recommended food habits, regardless of the SEC measure used. The strongest associations were found for participant’s own education, occupational class, and current financial difficulties. Participant’s own education, however, explained a considerable part of the associations between participant’s own occupational class and having several recommended food habits. Although adult SEC were more strongly associated with recommended food habits than childhood SEC, childhood SEC remained associated with having several recommended food habits after adjustment for participant’s own education, occupational class, and household income. Participants’ country of birth, marital status and children living in the household did not contribute to the associations of childhood and adult SEC with food habits. However, since only a minority of all participants (16% of women and 8% of men) had several recommended food habits, improvements in food habits are needed among all employees, including those with advantageous SEC.

### Interpretation of the findings

Of the single food habits, the found associations of more advantaged SEC with more frequent consumption of fruit and vegetables have been broadly supported in previous studies [7, 9, 10, 40]. In addition to quantity, the variety of consumed fruit and vegetables has been shown to be greater among individuals with more advantaged SEC [41]. Beyond fruit and vegetables, we found that consumption of skimmed milk products, fish, and vegetable-based cooking fat were socioeconomically patterned. A recent systematic review showed that more advantaged SEC, especially higher parental education, were associated with greater consumption of fruit and vegetables and dairy products, and lower consumption of sugary sweetened beverages and energy-dense foods among adolescents and young adults in high-income countries [9]. Another review found that fresh fruit and vegetables, whole grains, lean meats, fish, and low-fat dairy products were more likely to be consumed among individuals with more advantaged SEC, whereas refined grains and added fats were less likely to be consumed among these individuals [7]. Moreover, a systematic review on the Australian population showed that socioeconomically more advantaged groups were more likely to consume healthier food groups in general, but variations existed between and within studies depending on the SEC measure and food group used [40].

The inconsistent findings for fat spread in our study might be explained by the ‘modernity hypothesis’: although butter consumption has traditionally been higher among individuals with more disadvantaged SEC in Finland [11], the increasing selections of oil butter spreads (whose fat compositions are not as recommended) may attract more individuals with advantaged SEC who are more open to new and fashionable food products [2, 21]. Additionally, the inverse associations of housing tenure and household wealth with recommended red or processed meat consumption may suggest that individuals with greater wealth can afford to buy a variety of expensive meat products. However, other socioeconomic measures such as occupational class and parental and participant’s own education showed positive associations with recommended red or processed meat consumption, which is in line with a recent study on Finnish adults [12].

While there exist some heterogeneities in the associations between different SEC measures and single food habits, the associations between advantaged SEC and healthier overall diets have been consistent [9, 40]. These studies have used dietary patterns and scores, for instance, to measure diets more broadly [9, 40]. Our findings, which indicated that multiple adult SEC were associated with having several recommended food habits, are in line with our previous study on midlife employees [25]. In both studies, childhood SEC did not explain these associations. In contrast to the previous study [25], however, we found that more advantaged childhood SEC were also associated with having several recommended food habits, independently of conventional adult SEC measures. One probable explanation for this is that childhood is temporally closer in young than in midlife adults, thus, the impacts of childhood SEC on current health behaviours can be stronger for younger adults. Parental food habits and eating behaviours, which are socioeconomically patterned, commonly transfer to offspring [42, 43], and these probably mirror young adults’ food habits. Another difference between this study of younger employees and our previous study of midlife employees [25] is that in this study, participant’s own education explained most of the associations between occupational class and food habits, but not vice versa. Thus, educational attainment, which often precedes occupational class [24], seems to play a central role in how SEC are associated with younger adults’ food habits. A recent systematic review also found that education, more than occupational class and income, showed a clear association with overall diet among adolescents and young adults [9]. The effect of occupational class can possibly increase over time as employees are longer influenced by work-related characteristics such as working conditions [44].

The associations between material circumstances, especially current financial difficulties and household wealth, showed consistent and independent positive associations with both single food habits and the dichotomous ‘several recommended food habits’ variable. Financial difficulties, in particular, have shown a strong and consistent association with food habits in previous studies [4]. Subjective experiences of material challenges presumably affect the diversity of foods individuals buy, and consequently how nutritious their diets are. An Australian systematic review found that although socioeconomically disadvantaged groups spent less money on food than socioeconomically advantaged groups, they used proportionally more of their household budget on food [45]. Moreover, cost acts more often as a barrier to consuming healthy foods among individuals with disadvantaged SEC [46]. Diet cost has also been shown to mediate the pathway between income and diet quality [18]. Although individuals can experience financial difficulties across all socioeconomic groups, these problems have a larger influence on everyday food choices and eating practices of individuals with disadvantaged SEC [25, 47].

### Limitations and strengths

The 14-item FFQ provides only limited information on participants’ food and dietary habits. Portion sizes were not available, which limits the possibilities to make strong conclusions about the healthiness of participants’ diets. For instance, consumption of vegetables, fruit, or berries at least twice a day may not reach the recommended amount of at least 500 g a day [28]. However, we did not use a stricter criterion for the consumption of vegetables, fruit, and berries since few participants (12% of women and 5% of men) met this criterion (Table S10, Additional file 1). Supplementary analyses for women showed that the associations between SEC and recommended food habits were mostly similar or slightly stronger when using the stricter criterion (Table S11, Additional file 1). The FFQ did not enable us to estimate participants’ energy intake, which could have provided more information about the healthiness of their diets. Participants’ consumption of each food was based on self-reports, which are known to be affected by recall and social desirability biases [48, 49]. The ability to quantify consumed foods may also be less developed among individuals with disadvantaged SEC [10]. Additionally, retrospective data on childhood SEC may be influenced by recall bias, especially among individuals with disadvantaged SEC [50] and among older participants.

We analysed women and men together in our main analyses because of the small number of men in the study (and target) population, which disregards potential gender differences in the associations. A previous study of midlife employees of the City of Helsinki showed that the associations between SEC and food habits were mostly parallel but varied somewhat by gender [25]. For instance, participant’s occupational class showed stronger associations with healthy food habits among women than men, whereas home ownership and financial difficulties in adulthood showed stronger associations for men than women. However, we did not observe gender interaction in the associations between SEC measures and food habits. The supplementary analyses confirmed that the associations were mostly parallel between genders, though statistically significant associations were more often observed in women (Tables S3–S6, Additional file 1).

The response rate for the survey was 51.5%, and we further excluded participants with telephone interviews and with missing data on key variables (22%), which may produce selection bias. Non-respondents were more often men, manual workers, and from the lowest income quartile [27], thus it is possible that the socioeconomic gradient is stronger in the target population (see 2.1 Data and participants). However, our sensitivity analyses suggested that the participants in this study (*N* = 4621) did not differ from participants in the initial study sample (*N* = 5898) in terms of socioeconomic characteristics and vegetable consumption (Table S9, Additional file 1). Differences in the distributions of socioeconomic and health-related factors have also been shown to be small in general between the target population (*N* = 11,459) and the initial study sample (*N* = 5898) [27]. Thus, although the results are not generalisable to the general Finnish population (since the participants are municipal employees and mostly women), the data represent the target population reasonably well. Additionally, the large proportion of women (80%) in this study well corresponds to their proportion in the Finnish municipal sector.

Another strength of this paper is the use of multiple measures of both SEC and food habits, which provides a comprehensive view on socioeconomic differences in employees’ food habits. Research on socioeconomic differences in food habits in younger adults has been scarcer than in older adults [9], although younger adults are an important population group from the prevention point of view. For example, a previous study showed that most of adult weight gain occurred in early adulthood where socioeconomic differences in body weight already existed [51]. Thus, our findings provide useful and up-to-date information for employers and policy makers to plan targeted interventions to reduce socioeconomic differences in employees’ food habits. This is topical especially now as the COVID-19 pandemic is likely to further increase socioeconomic differences in food habits [20].

### Policy implications of the findings

Since our study showed that socioeconomic differences in employees’ food habits were consistent, regardless of the SEC measure used, policy actions should ensure that individuals with less material resources can afford diverse selections of healthy foods. Environmental changes to promote choosing healthy foods might be efficient; for instance, improving the availability of staff canteens in workplaces characterised by employees of lower socioeconomic groups could increase consumption of healthy foods among these employees [52, 53]. Additionally, targeted interventions to promote nutrition knowledge among individuals with disadvantaged SEC may increase healthy food choices among these individuals [6, 54]. Overall, improvements in material and structural factors (e.g., working conditions, food taxes, and subsidies) that consider sociocultural and cognitive aspects of adhering to healthy diets are needed among individuals with disadvantaged SEC, so that socioeconomic differences in food habits—and in health more broadly—can be diminished.

## Conclusions

The present study on 19–39-year-old Finnish municipal employees showed that employees with advantageous childhood and adult socioeconomic circumstances (SEC) were more likely to have several recommended food habits compared to those with more disadvantageous SEC. Additionally, more frequent consumption of fruit and vegetables and fish as well as preferring vegetable-based oils in cooking were observed among participants with more advantageous SEC, which is in line with previous national and international studies. Thus, socioeconomic differences in employees’ food habits persist, which may have unfavourable consequences in terms of future diet-related health disparities. Future studies should map the most effective interventions to tackle socioeconomic differences in employees’ food habits. Given that childhood SEC contribute to younger employees’ food habits as this study showed, future studies should investigate closer the intergenerational mechanisms that influence the adherence to recommended food habits.

## Supplementary Information

**Additional file 1: Table S1**. Associations between socioeconomic circumstances and having several recommended food habits ^a^: additionally adjusted analyses ^b^. **Table S2**. Gender-specific Spearman correlation coefficients ^a^ and variance inflation factors (VIF) for socioeconomic measures. **Table S3**. Age-adjusted associations between socioeconomic circumstances and single food habits among women (*N* = 3677). **Table S4**. Age-adjusted associations between socioeconomic circumstances and single food habits among men (*N* = 944). **Table S5**. Associations between socioeconomic circumstances and having several recommended food habits ^a^ among women (*N* = 3677). **Table S6**. Associations between socioeconomic circumstances and having several recommended food habits ^a^ among men (*N* = 944). **Table S7**. Gender-adjusted associations between socioeconomic circumstances and having several recommended food habits ^a^ by age groups. **Table S8**. Number of recommended food habits and their share among the study participants. **Table S9**. Consumption of fresh vegetables at least twice a day by respondents’ education and occupational class. **Table S10**. Distributions of having several recommended food habits among the study participants, using the stricter criterion ^a^. **Table S11**. Associations between socioeconomic circumstances and having several recommended food habits (with a ‘stricter criterion’) ^a^ among women (*N* = 3677). **Figure S1**. Flow chart of the study population.

## Data Availability

The dataset analysed during the current study is not publicly available but is available from the corresponding author on reasonable request. The Helsinki Health Study data are available exclusively to the researchers and will only be used for scientific purposes.

## Abbreviations

CI: Confidence interval
FFQ: Food frequency questionnaire
OECD: Organisation for Economic Co-operation and Development
OR: Odds ratio
SEC: Socioeconomic circumstances
VIF: Variance inflation factor
