# Supplementary material for: Associations of childhood and adult socioeconomic circumstances with recommended food habits among young and midlife Finnish employees
Source: BMC Nutr. 2022 Jul 14;8:65. doi: 10.1186/s40795-022-00557-0 (PMC9281257; doi:10.1186/s40795-022-00557-0)
Supplement: Supplementary file 1 — Additional file 1: Table S1. Associations between socioeconomic circumstances and having several recommended food habits a: additionally adjusted analyses b. Table S2. Gender-specific Spearman correlation coefficients a and variance inflation factors (VIF) for socioeconomic measures. Table S3. Age-adjusted associations between socioeconomic circumstances and single food habits among women (N = 3677). Table S4. Age-adjusted associations between socioeconomic circumstances and single food habits among men (N = 944). Table S5. Associations between socioeconomic circumstances and having several recommended food habits a among women (N = 3677). Table S6. Associations between socioeconomic circumstances and having several recommended food habits a among men (N = 944). Table S7. Gender-adjusted associations between socioeconomic circumstances and having several recommended food habits a by age groups. Table S8. Number of recommended food habits and their share among the study participants. Table S9. Consumption of fresh vegetables at least twice a day by respondents’ education and occupational class. Table S10. Distributions of having several recommended food habits among the study participants, using the stricter criterion a. Table S11. Associations between socioeconomic circumstances and having several recommended food habits (with a ‘stricter criterion’) a among women (N = 3677). Figure S1. Flow chart of the study population. [file 40795_2022_557_MOESM1_ESM.docx]

**Table S1.** Associations between socioeconomic circumstances and having several recommended food habits ^a^: additionally adjusted analyses ^b^.

|  | **Odds ratios (95% confidence intervals)** |
| --- | --- |
| ***Childhood socioeconomic measures*** |  |
| **Parental educational level ^c^** |  |
| Upper secondary school | 1.19 (0.90-1.56) |
| Higher education | 1.48 (1.23-1.77) |
| **Childhood financial difficulties ^d^** |  |
| No | 1.39 (1.12-1.73) |
| ***Adult socioeconomic measures*** |  |
| **Educational level ^e^** |  |
| Bachelor’s degree | 1.59 (1.27-1.99) |
| Master’s degree or higher | 2.14 (1.70-2.69) |
| **Occupational class ^f^** |  |
| Routine non-manual worker | 1.42 (0.80-2.54) |
| Semi-professional | 1.97 (1.12-3.46) |
| Manager or professional | 2.65 (1.50-4.68) |
| **Household income ^g^** |  |
| 2^nd^ lowest quartile | 1.22 (0.96-1.57) |
| 2^nd^ highest quartile | 1.43 (1.10-1.85) |
| Highest quartile | 1.86 (1.43-2.41) |
| **Housing tenure ^h^** |  |
| Owner-occupier | 1.32 (1.10-1.58) |
| **Financial difficulties ^i^** |  |
| No or few | 2.10 (1.65-2.68) |
| **Household wealth ^j^** |  |
| 10,000–99,999€ | 1.47 (1.19-1.82) |
| ≥100,000€ | 1.70 (1.34-2.16) |

^a^ Having 6–8 recommended food habits and consuming fresh or cooked vegetables, or fruit or berries at least twice a day was required for belonging to this group. Comparison group: having 0–5 recommended food habits.

^b^ The binary logistic regression analyses are adjusted for gender (woman / man), age (19–29 years/ 30–39 years), country of birth (Finland / other), marital status (married or cohabiting / other), having children in the household (no / yes), body mass index (BMI<30 kg/m^2^ / BMI≥30 kg/m^2^), and long-term illness (no / yes).

Reference groups: ^c^ vocational school or lower, ^d^ yes, ^e^ upper secondary school or lower, ^f^ manual worker, ^g^ lowest quartile, ^h^ renter or other, ^i^ yes, ^j^ <10,000€.

**Table S2.**  Gender-specific Spearman correlation coefficients ^a^ and variance inflation factors (VIF) for socioeconomic measures.

|  | ***Women VIF*** | **Parental educational level** | **Childhood financial difficulties** | **Own educational level** | **Occupational class** | **Household income** | **Housing tenure** | **Current financial difficulties** | **Household wealth** | ***Men VIF*** |
| --- | --- | --- | --- | --- | --- | --- | --- | --- | --- | --- |
| **Parental educational level** | *1.09* | *1* | 0.208* | 0.255* | 0.239* | 0.123* | -0.012 | 0.078* | 0.039 | *1.12* |
| **Childhood financial difficulties** | *1.04* | 0.135* | *1* | 0.144* | 0.085* | 0.117* | 0.086* | 0.228* | 1.182* | *1.11* |
| **Own educational level** | *2.76* | 0.262* | 0.088* | *1* | 0.720* | 0.317* | 0.086* | 0.228* | 0.182* | *2.12* |
| **Occupational class** | *2.63* | 0.232* | 0.083* | 0.800* | *1* | 0.279* | 0.048 | 0.192* | 0.114* | *2.02* |
| **Household income** | *1.25* | 0.120* | 0.069* | 0.348* | 0.340* | *1* | 0.150* | 0.316* | 0.267* | *1.24* |
| **Housing tenure** | *1.53* | 0.049* | 0.058* | 0.227* | 0.195* | 0.238* | *1* | 0.159* | 0.578* | *1.50* |
| **Current financial difficulties** | *1.14* | 0.083* | 0.123* | 0.245* | 0.228* | 0.256* | 0.129* | *1* | 0.299* | *1.20* |
| **Household wealth** | *1.69* | 0.119* | 0.137* | 0.294* | 0.244* | 0.316* | 0.580* | 0.264* | *1* | *1.69* |

^a^ Coefficients for women are shown below the diagonal, and for men, above the diagonal.

*Statistical significance level 0.05.

**Table S3.** Age-adjusted associations between socioeconomic circumstances and single food habits among women (N=3677).

|  | **Odds ratios (95% confidence intervals)** | | | | | | | |
| --- | --- | --- | --- | --- | --- | --- | --- | --- |
|  | **Fresh or cooked vegetables at least twice a day** | **Fruit or berries at least twice a day** | **Dark bread daily** | **Skimmed milk products daily** | **Fish at least 2–4 times/week** | **Red or processed meat 2–4 times/week at most** | **Vegetable-based margarine on bread** | **Vegetable-based margarine or oil in cooking** |
| ***Childhood socioeconomic measures*** | | | | | | | | |
| **Parental educational level ^a^** | | | | | | | | |
| Upper secondary school | 1.16 (0.94-1.43) | 1.12 (0.90-1.40) | 1.00 (0.81-1.24) | 1.03 (0.83-1.28) | 0.99 (0.77-1.27) | 1.49 (1.11-2.00) | 0.88 (0.70-1.09) | 1.33 (1.05-1.67) |
| Higher education | 1.42 (1.23-1.63) | 1.25 (1.08-1.44) | 1.03 (0.89-1.18) | 1.18 (1.02-1.36) | 1.44 (1.23-1.69) | 1.36 (1.13-1.64) | 0.94 (0.81-1.08) | 1.48 (1.27-1.73) |
| **Childhood financial difficulties ^b^** | | | | | | | | |
| No | 1.05 (0.89-1.23) | 1.20 (1.01-1.42) | 1.14 (0.97-1.35) | 1.20 (1.01-1.41) | 1.18 (0.98-1.42) | 1.22 (0.99-1.49) | 1.07 (0.90-1.26) | 1.09 (0.92-1.29) |
| ***Adult socioeconomic measures*** | | | | | | | | |
| **Educational level ^c^** |  |  |  |  |  |  |  |  |
| Bachelor’s degree | 1.52 (1.30-1.78) | 1.57 (1.33-1.86) | 1.25 (1.06-1.46) | 1.22 (1.04-1.44) | 1.18 (0.98-1.42) | 1.16 (0.95-1.42) | 0.85 (0.72-1.00) | 1.75 (1.48-2.07) |
| Master’s degree or higher | 1.88 (1.58-2.24) | 1.86 (1.55-2.23) | 1.10 (0.92-1.30) | 1.41 (1.18-1.68) | 1.57 (1.29-1.91) | 1.63 (1.29-2.07) | 0.73 (0.61-0.88) | 2.11 (1.75-2.55) |
| **Occupational class ^d^** |  |  |  |  |  |  |  |  |
| Routine non-manual worker | 1.55 (1.01-2.38) | 1.04 (0.67-1.63) | 1.57 (1.00-2.46) | 1.11 (0.71-1.73) | 0.87 (0.54-1.40) | 1.25 (0.76-2.06) | 0.83 (0.55-1.26) | 1.12 (0.74-1.70) |
| Semi-professional | 2.13 (1.40-3.25) | 1.30 (0.84-2.02) | 1.87 (1.21-2.91) | 1.49 (0.96-2.30) | 0.98 (0.62-1.55) | 1.32 (0.81-2.15) | 0.71 (0.47-1.06) | 1.94 (1.29-2.93) |
| Manager or professional | 2.83 (1.84-4.35) | 1.61 (1.03-2.52) | 1.63 (1.04-2.55) | 1.48 (0.95-2.31) | 1.24 (0.78-1.98) | 1.91 (1.15-3.18) | 0.66 (0.43-1.00) | 2.40 (1.57-3.67) |
| **Household income ^e^** |  |  |  |  |  |  |  |  |
| 2^nd^ lowest quartile | 1.06 (0.89-1.26) | 1.12 (0.93-1.34) | 0.95 (0.80-1.14) | 1.02 (0.85-1.22) | 0.93 (0.76-1.14) | 1.26 (1.00-1.59) | 1.06 (0.89-1.27) | 1.06 (0.88-1.27) |
| 2^nd^ highest quartile | 1.41 (1.17-1.70) | 1.41 (1.16-1.71) | 0.97 (0.81-1.17) | 1.06 (0.88-1.29) | 1.08 (0.87-1.33) | 1.20 (0.94-1.52) | 0.93 (0.77-1.13) | 1.54 (1.26-1.89) |
| Highest quartile | 1.60 (1.31-1.95) | 1.57 (1.28-1.93) | 0.97 (0.79-1.18) | 1.31 (1.07-1.60) | 1.35 (1.09-1.68) | 1.28 (0.99-1.67) | 0.97 (0.79-1.19) | 1.50 (1.21-1.87) |
| **Housing tenure ^f^** |  |  |  |  |  |  |  |  |
| Owner-occupiers | 1.35 (1.17-1.54) | 1.10 (0.95-1.26) | 1.18 (1.03-1.35) | 1.28 (1.12-1.48) | 1.33 (1.14-1.55) | 0.77 (0.64-0.92) | 1.09 (0.94-1.25) | 1.24 (1.07-1.44) |
| **Financial difficulties ^g^** |  |  |  |  |  |  |  |  |
| No or few | 1.43 (1.22-1.67) | 1.45 (1.22-1.71) | 1.23 (1.05-1.44) | 1.10 (0.93-1.29) | 1.39 (1.16-1.68) | 1.24 (1.01-1.51) | 1.06 (0.90-1.25) | 1.77 (1.50-2.09) |
| **Household wealth ^h^** |  |  |  |  |  |  |  |  |
| 10,000–99,999€ | 1.24 (1.07-1.44) | 1.26 (1.07-1.47) | 1.13 (0.97-1.31) | 1.14 (0.98-1.34) | 1.22 (1.02-1.45) | 0.97 (0.79-1.19) | 0.99 (0.85-1.16) | 1.38 (1.17-1.62) |
| ≥100,000€ | 1.52 (1.28-1.81) | 1.37 (1.15-1.64) | 1.21 (1.02-1.44) | 1.49 (1.24-1.77) | 1.66 (1.36-2.01) | 0.85 (0.68-1.07) | 1.16 (0.97-1.39) | 1.55 (1.28-1.88) |

Reference groups: ^a^ vocational school or lower, ^b^ yes, ^c^ upper secondary school or lower, ^d^ manual worker, ^e^ lowest quartile, ^f^ renter or other, ^g^ yes, ^h^ <10,000€.

**Table S4.** Age-adjusted associations between socioeconomic circumstances and single food habits among men (N=944).

|  | **Odds ratios (95% confidence intervals)** | | | | | | | |
| --- | --- | --- | --- | --- | --- | --- | --- | --- |
|  | **Fresh or cooked vegetables at least twice a day** | **Fruit or berries at least twice a day** | **Dark bread daily** | **Skimmed milk products daily** | **Fish at least 2–4 times/week** | **Red or processed meat 2–4 times/week at most** | **Vegetable-based margarine on bread** | **Vegetable-based margarine or oil in cooking** |
| ***Childhood socioeconomic measures*** | | | | | | | | |
| **Parental educational level ^a^** |  |  |  |  |  |  |  |  |
| Upper secondary school | 1.54 (0.99-2.39) | 1.10 (0.63-1.91) | 1.09 (0.71-1.67) | 0.98 (0.62-1.55) | 1.06 (0.66-1.68) | 1.14 (0.72-1.83) | 0.83 (0.55-1.27) | 1.00 (0.65-1.53) |
| Higher education | 1.52 (1.12-2.07) | 1.47 (1.02-2.12) | 1.02 (0.76-1.37) | 0.96 (0.71-1.32) | 1.39 (1.02-1.90) | 0.99 (0.72-1.35) | 0.79 (0.59-1.05) | 1.15 (0.86-1.55) |
| **Childhood financial difficulties ^b^** | | | | | | | | |
| No | 1.37 (0.97-1.93) | 1.17 (0.78-1.75) | 1.02 (0.74-1.40) | 1.24 (0.87-1.76) | 1.13 (0.81-1.59) | 0.89 (0.63-1.26) | 0.69 (0.51-0.93) | 0.79 (0.57-1.10) |
| ***Adult socioeconomic measures*** | | | | | | | | |
| **Educational level ^c^** |  |  |  |  |  |  |  |  |
| Bachelor’s degree | 1.50 (1.06-2.12) | 1.43 (0.95-2.15) | 1.25 (0.90-1.72) | 1.23 (0.87-1.76) | 1.55 (1.08-2.23) | 1.11 (0.79-1.56) | 0.86 (0.63-1.19) | 1.39 (1.01-1.93) |
| Master’s degree or higher | 1.95 (1.38-2.76) | 1.40 (0.92-2.12) | 0.93 (0.66-1.30) | 1.49 (1.05-2.13) | 2.69 (1.89-3.82) | 2.00 (1.37-2.93) | 0.91 (0.66-1.26) | 2.44 (1.67-3.44) |
| **Occupational class ^d^** |  |  |  |  |  |  |  |  |
| Routine non-manual worker | 1.90 (1.10-3.25) | 1.28 (0.72-2.28) | 1.33 (0.85-2.09) | 1.56 (0.94-2.60) | 0.91 (0.54-1.53) | 1.13 (0.72-1.77) | 0.78 (0.51-1.21) | 0.65 (0.41-1.02) |
| Semi-professional | 2.20 (1.30-3.71) | 1.30 (0.74-2.27) | 1.07 (0.69-1.67) | 1.24 (0.75-2.05) | 1.41 (0.87-2.30) | 1.66 (1.06-2.59) | 0.93 (0.61-1.41) | 0.85 (0.55-1.33) |
| Manager or professional | 2.95 (1.76-4.95) | 1.23 (0.70-2.16) | 1.07 (0.68-1.66) | 1.85 (1.13-3.03) | 2.20 (1.36-3.54) | 2.15 (1.36-3.40) | 0.75 (0.49-1.15) | 1.17 (0.74-1.84) |
| **Household income ^e^** |  |  |  |  |  |  |  |  |
| 2^nd^ lowest quartile | 1.28 (0.86-1.90) | 1.37 (0.87-2.16) | 1.30 (0.90-1.89) | 1.28 (0.86-1.90) | 1.18 (0.79-1.74) | 0.87 (0.60-1.28) | 0.80 (0.56-1.13) | 0.86 (0.60-1.24) |
| 2^nd^ highest quartile | 1.41 (0.94-2.12) | 1.15 (0.71-1.85) | 1.50 (1.03-2.19) | 1.13 (0.75-1.72) | 1.10 (0.73-1.66) | 1.20 (0.80-1.82) | 0.76 (0.53-1.10) | 1.19 (0.81-1.76) |
| Highest quartile | 1.96 (1.28-2.99) | 1.01 (0.59-1.71) | 0.97 (0.63-1.48) | 1.47 (0.95-2.28) | 1.71 (1.12-2.61) | 1.09 (0.70-1.70) | 0.77 (0.51-1.14) | 1.12 (0.74-1.70) |
| **Housing tenure ^f^** |  |  |  |  |  |  |  |  |
| Owner-occupier | 1.26 (0.94-1.69) | 1.16 (0.82-1.64) | 1.32 (0.99-1.75) | 1.18 (0.87-1.59) | 0.80 (0.59-1.08) | 0.80 (0.59-1.08) | 0.90 (0.68-1.19) | 1.05 (0.79-1.40) |
| **Financial difficulties ^g^** |  |  |  |  |  |  |  |  |
| No or few | 1.69 (1.17-2.45) | 1.25 (0.82-1.92) | 1.33 (0.95-1.87) | 1.58 (1.08-2.30) | 1.14 (0.80-1.62) | 1.00 (0.70-1.42) | 0.89 (0.64-1.22) | 1.67 (1.21-2.30) |
| **Household wealth ^h^** |  |  |  |  |  |  |  |  |
| 10,000–99,999€ | 1.19 (0.84-1.68) | 1.01 (0.68-1.50) | 1.31 (0.94-1.81) | 1.35 (0.94-1.94) | 1.06 (0.75-1.50) | 0.87 (0.61-1.24) | 0.97 (0.70-1.33) | 1.08 (0.79-1.49) |
| ≥100,000€ | 1.53 (1.04-2.26) | 0.84 (0.53-1.36) | 1.29 (0.88-1.88) | 1.84 (1.23-2.76) | 1.48 (1.00-2.19) | 0.80 (0.53-1.19) | 1.24 (0.86-1.80) | 1.28 (0.87-1.88) |

Reference groups: ^a^ vocational school or lower, ^b^ yes, ^c^ upper secondary school or lower, ^d^ manual worker, ^e^ lowest quartile, ^f^ renter or other, ^g^ yes, ^h^ <10,000€.

**Table S5.** Associations between socioeconomic circumstances and having several recommended food habits ^a^ among women (N=3677).

|  | **Odds ratios (95% confidence intervals)** | | | |
| --- | --- | --- | --- | --- |
|  | **M1**: Age-adjustment | **M2**: M1 + country of birth, marital status, and having children in household | **M3 ^b^**: M2 + parental educational level and childhood financial difficulties | **M4 ^b^**: M2 + own education, occupational class, and household income |
| ***Childhood socioeconomic measures*** | | | | |
| **Parental educational level ^c^** | | | | |
| Upper secondary school | 1.20 (0.90-1.60) | 1.19 (0.89-1.59) | 1.18 (0.88-1.58) | 1.12 (0.84-1.50) |
| Higher education | 1.47 (1.22-1.77) | 1.48 (1.22-1.79) | 1.43 (1.18-1.73) | 1.26 (1.03-1.54) |
| **Childhood financial difficulties ^d^** | | | | |
| No | 1.45 (1.15-1.83) | 1.45 (1.15-1.83) | 1.38 (1.09-1.74) | 1.35 (1.07-1.71) |
| ***Adult socioeconomic measures*** | | | | |
| **Educational level ^e^** |  |  |  |  |
| Bachelor’s degree | 1.61 (1.28-2.04) | 1.61 (1.27-2.04) | 1.54 (1.21-1.95) | 1.47 (1.06-2.04) |
| Master’s degree or higher | 2.15 (1.69-2.74) | 2.18 (1.70-2.78) | 1.96 (1.52-2.54) | 1.85 (1.24-2.75) |
| **Occupational class ^f^** |  |  |  |  |
| Routine non-manual worker | 1.37 (0.67-2.80) | 1.38 (0.68-2.83) | 1.38 (0.67-2.81) | 1.45 (0.71-2.97) |
| Semi-professional | 2.04 (1.01-4.10) | 2.02 (1.00-4.06) | 1.93 (0.96-3.89) | 1.54 (0.74-3.18) |
| Manager or professional | 2.67 (1.32-5.38) | 2.68 (1.33-5.42) | 2.40 (1.18-4.87) | 1.57 (0.74-3.37) |
| **Household income ^g^** |  |  |  |  |
| 2^nd^ lowest quartile | 1.03 (0.80-1.32) | 1.16 (0.90-1.51) | 1.12 (0.86-1.45) | 1.02 (0.78-1.33) |
| 2^nd^ highest quartile | 1.32 (1.02-1.69) | 1.40 (1.07-1.85) | 1.33 (1.01-1.75) | 1.17 (0.88-1.55) |
| Highest quartile | 1.55 (1.19-2.00) | 1.75 (1.33-2.31) | 1.62 (1.22-2.14) | 1.35 (1.00-1.81) |
| **Housing tenure ^h^** |  |  |  |  |
| Owner-occupier | 1.43 (1.19-1.71) | 1.30 (1.07-1.57) | 1.24 (1.02-1.50) | 1.14 (0.93-1.39) |
| **Financial difficulties ^i^** |  |  |  |  |
| No or few | 2.09 (1.63-2.69) | 2.11 (1.63-2.72) | 2.00 (1.55-2.58) | 1.81 (1.39-2.35) |
| **Household wealth ^j^** |  |  |  |  |
| 10,000–99,999€ | 1.50 (1.21-1.86) | 1.45 (1.16-1.81) | 1.38 (1.10-1.72) | 1.28 (1.02-1.61) |
| ≥100,000€ | 1.81 (1.43-2.29) | 1.65 (1.29-2.11) | 1.49 (1.15-1.91) | 1.33 (1.02-1.73) |

^a^ Having 6–8 recommended food habits and consuming fresh or cooked vegetables, or fruit or berries at least twice a day was required for belonging to the group of having several recommended food habits. Comparison group: having 0–5 recommended food habits.

^b^ Mutual adjustment has been performed in Models 3 and 4 in the cases where there are same variables both as an exposure and as a covariate. For example, concerning parental educational level as an exposure measure in Model 3, the analysis is adjusted for Model 2 covariates together with childhood financial difficulties.

Reference groups: ^c^ vocational school or lower, ^d^ yes, ^e^ upper secondary school or lower, ^f^ manual worker, ^g^ lowest quartile, ^h^ renter or other, ^i^ yes, ^j^ <10,000€.

**Table S6.** Associations between socioeconomic circumstances and having several recommended food habits ^a^ among men (N=944).

|  | **Odds ratios (95% confidence intervals)** | | | |
| --- | --- | --- | --- | --- |
|  | **M1**: Age-adjustment | **M2**: M1 + country of birth, marital status, and having children in household | **M3 ^b^**: M2 + parental educational level and childhood financial difficulties | **M4 ^b^**: M2 + own education, occupational class, and household income |
| ***Childhood socioeconomic measures*** | | | | |
| **Parental educational level ^c^** | | | | |
| Upper secondary school | 1.30 (0.58-2.89) | 1.26 (0.56-2.82) | 1.25 (0.56-2.81) | 1.12 (0.49-2.54) |
| Higher education | 1.71 (1.00-2.92) | 1.75 (1.02-3.00) | 1.73 (1.00-3.00) | 1.37 (0.77-2.42) |
| **Childhood financial difficulties ^d^** | | | | |
| No | 1.23 (0.68-2.22) | 1.18 (0.65-2.15) | 1.06 (0.57-1.95) | 0.98 (0.53-1.81) |
| ***Adult socioeconomic measures*** | | | | |
| **Educational level ^e^** |  |  |  |  |
| Bachelor’s degree | 1.45 (0.76-2.75) | 1.51 (0.79-2.88) | 1.42 (0.74-2.73) | 1.42 (0.66-3.05) |
| Master’s degree or higher | 2.44 (1.35-4.41) | 2.48 (1.37-4.51) | 2.23 (1.19-4.17) | 1.85 (0.73-4.68) |
| **Occupational class ^f^** |  |  |  |  |
| Routine non-manual worker | 1.86 (0.66-5.19) | 1.87 (0.67-5.26) | 1.82 (0.65-5.13) | 1.76 (0.62-4.96) |
| Semi-professional | 1.65 (0.60-4.57) | 1.70 (0.61-4.73) | 1.59 (0.57-4.46) | 1.27 (0.41-3.92) |
| Manager or professional | 3.28 (1.25-8.64) | 3.21 (1.21-8.48) | 2.82 (1.05-7.59) | 1.67 (0.50-5.62) |
| **Household income ^g^** |  |  |  |  |
| 2^nd^ lowest quartile | 1.44 (0.71-2.95) | 1.70 (0.81-3.56) | 1.66 (0.79-3.50) | 1.46 (0.68-3.12) |
| 2^nd^ highest quartile | 1.31 (0.62-2.77) | 1.52 (0.70-3.27) | 1.49 (0.69-3.22) | 1.26 (0.57-2.77) |
| Highest quartile | 2.24 (1.09-4.60) | 2.70 (1.28-5.71) | 2.47 (1.16-5.27) | 1.92 (0.87-4.28) |
| **Housing tenure ^h^** |  |  |  |  |
| Owner-occupier | 1.55 (0.93-2.58) | 1.32 (0.77-2.28) | 1.32 (0.76-2.27) | 1.20 (0.68-2.10) |
| **Financial difficulties ^i^** |  |  |  |  |
| No or few | 2.33 (1.10-4.94) | 2.38 (1.11-5.12) | 2.27 (1.05-4.92) | 1.80 (0.80-4.03) |
| **Household wealth ^j^** |  |  |  |  |
| 10,000–99,999€ | 1.73 (0.88-3.43) | 1.66 (0.83-3.32) | 1.61 (0.80-3.24) | 1.46 (0.72-2.96) |
| ≥100,000€ | 2.64 (1.29-5.40) | 2.24 (1.07-4.71) | 2.21 (1.04-4.73) | 1.71 (0.77-3.77) |

^a^ Having 6–8 recommended food habits and consuming fresh or cooked vegetables, or fruit or berries at least twice a day was required for belonging to the group of having several recommended food habits. Comparison group: having 0–5 recommended food habits.

^b^ Mutual adjustment has been performed in Models 3 and 4 in the cases where there are same variables both as an exposure and as a covariate. For example, concerning parental educational level as an exposure measure in Model 3, the analysis is adjusted for Model 2 covariates together with childhood financial difficulties.

Reference groups: ^c^ vocational school or lower, ^d^ yes, ^e^ upper secondary school or lower, ^f^ manual worker, ^g^ lowest quartile, ^h^ renter or other, ^i^ yes, ^j^ <10,000€.

**Table S7.** Gender-adjusted associations between socioeconomic circumstances and having several recommended food habits ^a^ by age groups.

|  | **Odds ratios (95% confidence intervals)** | |
| --- | --- | --- |
|  | **19–29-year-old employees**  **(n=1479)** | **30–39-year-old employees**  **(n=3142)** |
| ***Childhood socioeconomic measures*** | | |
| **Parental educational level ^b^** |  |  |
| Upper secondary school | 0.64 (0.35-1.19) | 1.46 (1.08-1.98) |
| Higher education | 1.32 (0.92-1.89) | 1.54 (1.26-1.89) |
| **Childhood financial difficulties ^c^** |  |  |
| No | 1.35 (0.84-2.17) | 1.44 (1.13-1.83) |
| ***Adult socioeconomic measures*** | | |
| **Educational level ^d^** |  |  |
| Bachelor’s degree | 1.98 (1.32-2.97) | 1.44 (1.11-1.87) |
| Master’s degree or higher | 3.33 (2.10-5.27) | 1.91 (1.48-2.47) |
| **Occupational class ^e^** |  |  |
| Routine non-manual worker | 4.75 (0.64-35.54) | 1.21 (0.65-2.25) |
| Semi-professional | 8.42 (1.14-61.95) | 1.56 (0.86-2.83) |
| Manager or professional | 14.05 (1.89-104.50) | 2.05 (1.13-3.73) |
| **Household income ^f^** |  |  |
| 2^nd^ lowest quartile | 1.16 (0.72-1.88) | 1.04 (0.79-1.36) |
| 2^nd^ highest quartile | 1.51 (0.93-2.46) | 1.26 (0.95-1.65) |
| Highest quartile | 2.28 (1.36-3.85) | 1.47 (1.11-1.93) |
| **Housing tenure ^g^** |  |  |
| Owner-occupier | 1.20 (0.83-1.73) | 1.52 (1.25-1.85) |
| **Financial difficulties ^h^** |  |  |
| No or few | 4.47 (2.33-8.61) | 1.80 (1.38-2.33) |
| **Household wealth ^i^** |  |  |
| 10,000–99,999€ | 1.36 (0.94-1.97) | 1.58 (1.23-2.02) |
| ≥100,000€ | 1.90 (1.19-3.05) | 1.91 (1.48-2.47) |

^a^ Having 6–8 recommended food habits and consuming fresh or cooked vegetables, or fruit or berries at least twice a day was required for belonging to this group. Comparison group: having 0–5 recommended food habits.

Reference groups: ^b^ vocational school or lower, ^c^ yes, ^d^ upper secondary school or lower, ^e^ manual worker, ^f^ lowest quartile, ^g^ renter or other, ^h^ yes, ^i^ <10,000€.

**Table S8.** Number of recommended food habits and their share among the study participants.

| **Number of recommended food habits** | **All,**  **n (%)** | **Women,**  **n (%)** | **Men,**  **n (%)** |
| --- | --- | --- | --- |
| 0 | 48 (1.0) | 30 (0.8) | 18 (1.9) |
| 1 | 354 (7.7) | 240 (6.5) | 114 (12.1) |
| 2 | 787 (17.0) | 574 (15.6) | 213 (22.6) |
| 3 | 1013 (21.9) | 765 (20.8) | 248 (26.7) |
| 4 | 976 (21.1) | 796 (21.7) | 180 (19.1) |
| 5 | 751 (16.3) | 657 (17.9) | 94 (10.0) |
| 6 | 464 (10.0) | 410 (11.2) | 54 (5.7) |
| 7 | 185 (4.0) | 168 (4.6) | 17 (1.8) |
| 8 | 43 (0.9) | 37 (1.0) | 6 (0.6) |

**Table S9.** Consumption of fresh vegetables at least twice a day by respondents’ education and occupational class.

|  | **All respondents,**  **N=5898;**  **n (%)** | **Chi2-test** | **Analytical sample,**  **N=4621;**  **n (%)** | **Chi2-test** |
| --- | --- | --- | --- | --- |
| **Educational level** |  | <0.001 |  | <0.001 |
| Upper secondary school or lower | 637 (30.4) |  | 485 (31.5) |  |
| Bachelor’s degree | 890 (42.8) |  | 739 (43.3) |  |
| Master’s degree or higher | 769 (45.8) |  | 621 (45.4) |  |
| **Occupational class** |  | <0.001 |  | <0.001 |
| Manual worker | 66 (19.6) |  | 41 (17.3) |  |
| Routine non-manual worker | 558 (34.7) |  | 427 (34.9) |  |
| Semi-professional | 943 (42.4) |  | 802 (42.9) |  |
| Manager or professional | 693 (45.1) |  | 575 (44.8) |  |

**Table S10.** Distributions of having several recommended food habits among the study participants, using the stricter criterion ^a^.

| **Number of recommended food habits** | **All, n (%)** | **Women, n (%)** | **Men, n (%)** |
| --- | --- | --- | --- |
| 0–5 (‘other’) | 4138 (89.6) | 3237 (88.0) | 901 (95.4) |
| 6–8 (‘having several recommended food habits’) | 483 (10.5) | 604 (12.0) | 43 (4.6) |

^a^ Having 6–8 recommended food habits and consuming fresh or cooked vegetables, and fruit or berries at least twice a day was required for belonging to the group of having several recommended food habits.

**Table S11.** Associations between socioeconomic circumstances and having several recommended food habits (with a ‘stricter criterion’) ^a^ among women (N=3677).

|  | **Odds ratios (95% confidence intervals)** | | | |
| --- | --- | --- | --- | --- |
|  | **M1**: Age-adjustment | **M2**: M1 + country of birth, marital status, and having children in household | **M3 ^b^**: M2 + parental educational level and childhood financial difficulties | **M4 ^b^**: M2 + own education, occupational class, and household income |
| ***Childhood socioeconomic measures*** | | | | |
| **Parental educational level ^c^** | | | | |
| Upper secondary school | 1.03 (0.74-1.45) | 1.02 (0.73-1.44) | 1.02 (0.72-1.43) | 0.95 (0.67-1.33) |
| Higher education | 1.37 (1.11-1.70) | 1.38 (1.11-1.71) | 1.32 (1.07-1.64) | 1.12 (0.90-1.41) |
| **Childhood financial difficulties ^d^** | | | | |
| No | 1.58 (1.21-2.08) | 1.58 (1.20-2.08) | 1.52 (1.15-2.00) | 1.47 (1.11-1.94) |
| ***Adult socioeconomic measures*** | | | | |
| **Educational level ^e^** |  |  |  |  |
| Bachelor’s degree | 1.83 (1.38-2.43) | 1.82 (1.37-2.41) | 1.76 (1.32-2.34) | 1.87 (1.27-2.74) |
| Master’s degree or higher | 2.62 (1.97-3.48) | 2.62 (1.96-3.49) | 2.45 (1.81-3.31) | 2.77 (1.76-4.36) |
| **Occupational class ^f^** |  |  |  |  |
| Routine non-manual worker | 1.20 (0.54-2.67) | 1.20 (0.54-2.69) | 1.19 (0.53-2.66) | 1.33 (0.59-2.99) |
| Semi-professional | 1.84 (0.84-4.03) | 1.81 (0.83-3.97) | 1.74 (0.79-3.81) | 1.23 (0.54-2.79) |
| Manager or professional | 2.46 (1.12-5.40) | 2.44 (1.11-5.37) | 2.21 (1.00-4.89) | 1.12 (0.48-2.63) |
| **Household income ^g^** |  |  |  |  |
| 2^nd^ lowest quartile | 0.99 (0.74-1.32) | 1.11 (0.82-1.49) | 1.06 (0.79-1.44) | 0.94 (0.69-1.28) |
| 2^nd^ highest quartile | 1.42 (1.07-1.89) | 1.48 (1.08-2.02) | 1.40 (1.03-1.91) | 1.19 (0.86-1.64) |
| Highest quartile | 1.52 (1.13-2.05) | 1.68 (1.22-2.31) | 1.56 (1.13-2.15) | 1.23 (0.88-1.72) |
| **Housing tenure ^h^** |  |  |  |  |
| Owner-occupier | 1.50 (1.22-1.85) | 1.37 (1.10-1.70) | 1.31 (1.05-1.63) | 1.20 (0.95-1.50) |
| **Financial difficulties ^i^** |  |  |  |  |
| No or few | 2.36 (1.74-3.19) | 2.34 (1.73-3.19) | 2.21 (1.63-3.01) | 1.98 (1.44-2.72) |
| **Household wealth ^j^** |  |  |  |  |
| 10,000–99,999€ | 1.67 (1.30-2.16) | 1.62 (1.25-2.11) | 1.54 (1.18-2.00) | 1.42 (1.09-1.86) |
| ≥100,000€ | 2.17 (1.65-2.84) | 2.00 (1.50-2.66) | 1.81 (1.35-2.42) | 1.60 (1.18-2.17) |

^a^ The stricter criterion for belonging to the group of having several recommended food habits was used: having 6–8 recommended food habits and consuming fresh or cooked vegetables, and fruit or berries at least twice a day was required. Comparison group: having 0–5 recommended food habits.

^b^ Mutual adjustment has been performed in Models 3 and 4 in the cases where there are same variables both as an exposure and as a covariate. For example, concerning parental educational level as an exposure measure in Model 3, the analysis is adjusted for Model 2 covariates together with childhood financial difficulties.

Reference groups: ^c^ vocational school or lower, ^d^ yes, ^e^ upper secondary school or lower, ^f^ manual worker, ^g^ lowest quartile, ^h^ renter or other, ^i^ yes, ^j^ <10,000€.


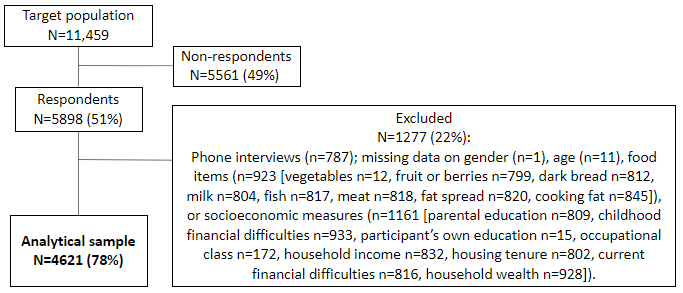


**Figure S1.** Flow chart of the study population.
